# MicroCT imaging applied to description of a new species of *Pagurus* Fabricius, 1775 (Crustacea: Decapoda: Anomura: Paguridae), with selection of three-dimensional type data

**DOI:** 10.1371/journal.pone.0203107

**Published:** 2018-09-26

**Authors:** Jannes Landschoff, Tomoyuki Komai, Anton du Plessis, Gavin Gouws, Charles L. Griffiths

**Affiliations:** 1 Department of Biological Sciences and Marine Research Institute, University of Cape Town, Rondebosch, Western Cape, South Africa; 2 Natural History Museum and Institute, Aoba-cho, Chuo-ku, Chiba, Japan; 3 CT Scanner, Central Analytical Facility, Stellenbosch University, Stellenbosch, Western Cape, South Africa; 4 National Research Foundation–South African Institute for Aquatic Biodiversity, Grahamstown, Eastern Cape, South Africa; Nanjing Agricultural University, CHINA

## Abstract

A new species of hermit crab, *Pagurus fraserorum* n. sp. (family Paguridae) is described from rocky subtidal reefs off KwaZulu-Natal, South Africa, and illustrated using both conventional drawings and colour photographs, and via three-dimensional (3D) X-ray micro-computed tomography (μCT). Because of the limitation μCT has in detecting very fine and soft structures, a novel approach of manually drawing setation and spinulation onto the two-dimensional images of the 3D visualizations was used to illustrate the pereopods. In addition, an interactive figure and rotation movie clips in the supplement section complement the species description, and the 3D raw data of the 3D type data are downloadable from the Gigascience Database repository. The new species is the sixth species of *Pagurus* Fabricius, 1775 reported from South Africa and is closely allied to the Indo-Pacific *P*. *boriaustraliensis* Morgen, 1990 and *P*. *pitagsaleei* McLaughlin, 2002, from which it differs by its shorter ocular peduncles, by the armature of the carpus of the right cheliped, and also in colouration. This study presents the first description of a hermit crab in which a majority of taxonomic details are illustrated through 3D volume-rendered illustrations. In addition, colour photographs and COI molecular barcodes are provided, and the latter compared to COI sequences of specimens from Western Australia previously identified as *P*. *boriaustraliensis* and of specimens of *P*. *pitagsaleei* from Taiwan, as well as to three additional South African members of the genus. The South African taxon was confirmed to be genetically distinct from all species tested.

## Introduction

As one of the most powerful tools for generating three-dimensional (3D) illustrations, X-ray micro-computed tomography (μCT) is gaining popularity in taxonomy and morphological studies of various invertebrate animal groups, such as planarians [1], oligochaetes [2], insects [3–6], arachnids [7], myriapods [8,9], and gammarid amphipods [10]. It has also been applied to marine invertebrates including sponges [11], cnidarians [12], polychaetes [13–15], rhizocephalans [16], bivalves [11], ophiuroids [17,18], and echinoids [19]. In higher taxa of Crustacea, the technique has been used to study sexual reproductive organs or internal anatomy [20–22], such as the lung complexity of terrestrial fiddler crabs [23], or the vascular system and inner anatomy in Anomura [24–26]. The latter, for example, brought new insights into the evolution of the hermit crab-derived king crabs. Nevertheless, μCT has not seen many applications in taxonomy of higher crustaceans, although Landschoff & Lemaitre [27] and Landschoff & Rahayu [28] included single μCT images or videos of calcified characters to inform the species description of paguroids, commonly known as hermit crabs. In this study, the discovery of a new hermit crab from South Africa is utilized to apply and test the technique of μCT for the description of a new decapod crustacean species.

With over 170 species worldwide the genus *Pagurus* Fabricius, 1775 [29] is the most species-rich genus in the hermit crab family Paguridae [30], although the heterogeneous nature of the genus has been pointed out (e.g. see Results in [31–33]. Of all South African paguroids (*sensu* McLaughlin et al. [34]) the genus *Pagurus* is one of the taxonomically best known. McLaughlin & Forest [35] reviewed earlier accounts of South African species (reported as *Eupagurus* Brand, 1851 [36] by Stebbing [37–39] and by Odhner [40], as well as the eight species listed by Barnard [41], seven as *Eupagurus* and one as ‘*incertae sedis*’). Moreover, those authors used newly-available material, primarily collected during the 1982–1986 R.V. *Meiring Naudé* cruises on the east coast, to take up questions about the true identifications of South African species raised by Forest [42]. They came to the conclusion that five species of *Pagurus* occur in South African waters, *P*. *carvicarpus* (Paul’son, 1875) [43], *P*. *cuanensis* Bell, 1845 [44], *P*. *emmersoni* McLaughlin & Forest, 1999 [35], *P*. *liochele* (Barnard, 1947) [45], and *P*. *prideaux* Leach, 1815 [46], plus a sixth undescribed species, of which only a single juvenile male had been found.

Recent SCUBA collections from the east coast of South Africa revealed another species assignable to *Pagurus* that was common on subtidal reefs off Pumula and Hibberdene, KwaZulu-Natal. This species is clearly not referable to any known South African species, but shows certain similarities to *P*. *boriaustraliensis* Morgan, 1990 [47], and *P*. *pitagsaleei* McLaughlin, 2002 [48]. It is herein fully described as new to science and compared with the latter two. For the first time in hermit crab taxonomy, a new species is illustrated by 3D μCT imaging techniques for major body parts, using 3D-visualizations in interactive portable document format (PDF), rotation videos, and 3D still images, some of which are also overlaid with manually drawn setae and corneous spines. In addition, full colour information as well as molecular barcodes are provided, and the latter are compared to DNA sequences of three other South African species (*P*. *cuanensis*, *P*. *emmersoni*, and *P*. *liochele*), as well as to sequences of the morphologically-similar *P*. *boriaustraliensis* from Western Australia and *P*. *pitagsaleei* from Taiwan.

## Material and methods

### Sampling and photography

Sampling and the handling of all biological samples were carried out under the University of Cape Town Science Faculty collection permit, as well as Animal Ethics Committee approval, protocol number 2014/DC1/CLG. Specimens of *Pagurus fraserorum* n. sp. were collected during two days of diving on near-shore reefs off Pumula and Hibberdene, approximately 100 km south of Durban, KwaZulu-Natal, South Africa. Back on land, the specimens were photographed alive in a photographic tank, then anesthetized in a 0.125 ml/l clove-oil seawater solution and frozen. After defrosting and extraction from their shells, colour images of the whole animal were taken in the laboratory of the University of Cape Town (UCT). All specimens were then preserved in 96% ethanol. The South African specimens of *P*. *cuanensis*, *P*. *emmersoni*, and *P*. *liochele* used for genetic comparison were collected on various sampling occasions in 2015–2016 (see specimen data in section of genetic comparison). Specimens are deposited at the Iziko South African Museum in Cape Town (SAMC) with two paratypic specimens also deposited at the Natural History Museum and Institute, Chiba, Japan (CBM). Two Taiwanese specimens of *Pagurus pitagsaleei* used for genetic comparison are located in the collections of the National Taiwan Ocean University, Keelung (NTOU). Comparative material previously identified as *P*. *boriaustraliensis* for physical examination and barcoding was also loaned from the Western Australian Museum (WAM). Measurements of specimens are given for shield length (SL) in millimetres (mm), taken from the tip of the rostrum to the midpoint of the posterior margin of the shield.

### MicroCT scanning and illustrations

X-ray micro-computed tomography (μCT) scans were performed at the CT Scanner Facility at Stellenbosch University, South Africa [49]. Two specimens of *P*. *fraserorum* n. sp. were scanned in three scans, using a General Electric Phoenix Nanotom S (Wunstorff, Germany). The holotype (male 2.7 mm, SAMC MB-A066790) was placed on rigid foam in a small container filled with ethanol and scanned whole at 11 μm voxel resolution. Because the left cheliped had broken off during previously handling, a 4.5 μm resolution scan of the left cheliped alone was also obtained applying the same method as before, but in order to obtain a higher magnification, the sample has to be positioned closer to the X-ray source of the scanner, hence a smaller container was used. Later, in the rendering of the computer software, the two scans were overlaid, putting the left cheliped virtually back into its place. In addition, a paratype (ovig. female 2.4 mm, SAMC MB-A066770) was wrapped in parafilm (Bemis NA, Neenah, WI, United States), mounted on rigid foam, which itself was glued to the top of a plastic rod, and scanned at 5 μm voxel resolution. In a datanote, Landschoff et al. [50] give detailed information on data acquisition and processing for species descriptions.

The 3D visualizations were carried out using Volume Graphics VGStudioMax 3.0. (Heidelberg, Germany). As described in du Plessis et al. [15] various parameters had to be optimized to obtain high quality data. Data processing was achieved using a combination of isosurface and Phong rendering. To remove noise from the 3D rendered images, a first-level surface determination was used, followed by an opening/closing function applied to the region of interest determined as the surface. Cropping removed unwanted material, often most efficiently used by applying the Polyline3D function from various angles. Moreover, this function was used for separation of the chelipeds and pereopods from the whole animal. Contrary to most microCT data, image filtration did not improve the visualizations, and especially setae and fine textures were lost when filtering was attempted. The process to select the region of interest for visualization as described above, could be continued with many more iterations of fine improvements, but with little improvement to the overall image. Hence, a good compromise was found between quality of the image and the processing time used.

Once selected, the object of interest was exported in the form of Stereolithography (STL) data, typically used for 3D printing. By loading these data into free software called MeshLab (http://www.meshlab.net), the data were converted to a Universal 3D (U3D) compressed format to be embedded in Adobe PDF files, or viewed online as interactive 3D files.

Drawings were initially made under a M5 Wild stereomicroscope with attached camera lucida. Pencil drawings were then digitally traced using inkscape (http://www.inkscape.com). To overcome the problem of visualizing fine and soft characters using μCT, the 3D rendered surface images of the μCT scan of the pereopods were overlaid with manual drawings of setae and spinules of the same sample. All figures were assembled in Gimp 2.8 (http://www.gimp.org).

### Genetic comparison

Genetic barcodes of the mitochondrial cytochrome *c* oxidase subunit I (COI) fragment were generated at the Aquatic Genomics Research Platform of the National Research Foundation—South African Institute for Aquatic Biodiversity, Grahamstown, using a methodology described previously [27]. Tissue was either taken from eggs of ovigerous females or the chelae and carpus of the right cheliped, and, for small specimens, from the coxae and ischia of different pereopods. DNA extraction, PCR amplification of the COI mitochondrial DNA fragment, purification of amplicons, fluorescently-labelled terminator cycle sequencing and sequence analysis proceeded as documented by Landschoff & Lemaitre [27]. One specimen each of *P*. *cuanensis* and *P*. *liochele* were barcoded by the Canadian Centre for DNA Barcoding, Biodiversity Institute of Ontario, University of Guelph, Canada. Sequences of the two Taiwanese specimens of *P*. *pitagsaleei* were kindly made available by Prof. Dr. Tin-Yam Chan (Institute of Marine Biology, National Taiwan Ocean University). Sequences of the South African and Indo-West Pacific specimens were uploaded to the SeaKeys (SEAKY) and Global Marine Crustacea Decapoda (GMDEC) projects on BOLD [51], and were submitted to GenBank. All COI barcodes were aligned in ClustalX 2.1 [52] and subsequently analyzed in MEGA7 [53], where a Neighbor-Joining [54] tree was constructed and nodal support was determined using 1000 bootstrap replicates [55]. Pairwise distances, corrected using a Kimura [56] 2-parameter (K2P) model of sequence evolution, were calculated between individuals and species.

### Nomenclatural acts

The electronic edition of this article conforms to the requirements of the amended International Code of Zoological Nomenclature, and hence the new names contained herein are available under that Code from the electronic edition of this article. This published work and the nomenclatural acts it contains have been registered in ZooBank, the online registration system for the ICZN. The ZooBank LSIDs (Life Science Identifiers) can be resolved and the associated information viewed through any standard web browser by appending the LSID to the prefix "http://zoobank.org/". The LSID for this publication is: urn:lsid:zoobank.org:pub: 20CC0F79-EC25-43FB-907D-F9A3B949FD93. The electronic edition of this work was published in a journal with an ISSN, and has been archived and is available from the following digital repositories: PubMed Central, LOCKSS.

## Systematics

### Taxonomy

Family Paguridae Latreille, 1802

Genus *Pagurus* Fabricius, 1775

*Pagurus fraserorum* Landschoff & Komai, **n. sp.**

urn:lsid:zoobank.org:act:F2D9994A-D7D1-469D-8FF8-DD9BE74896F3

**Fig 1:**
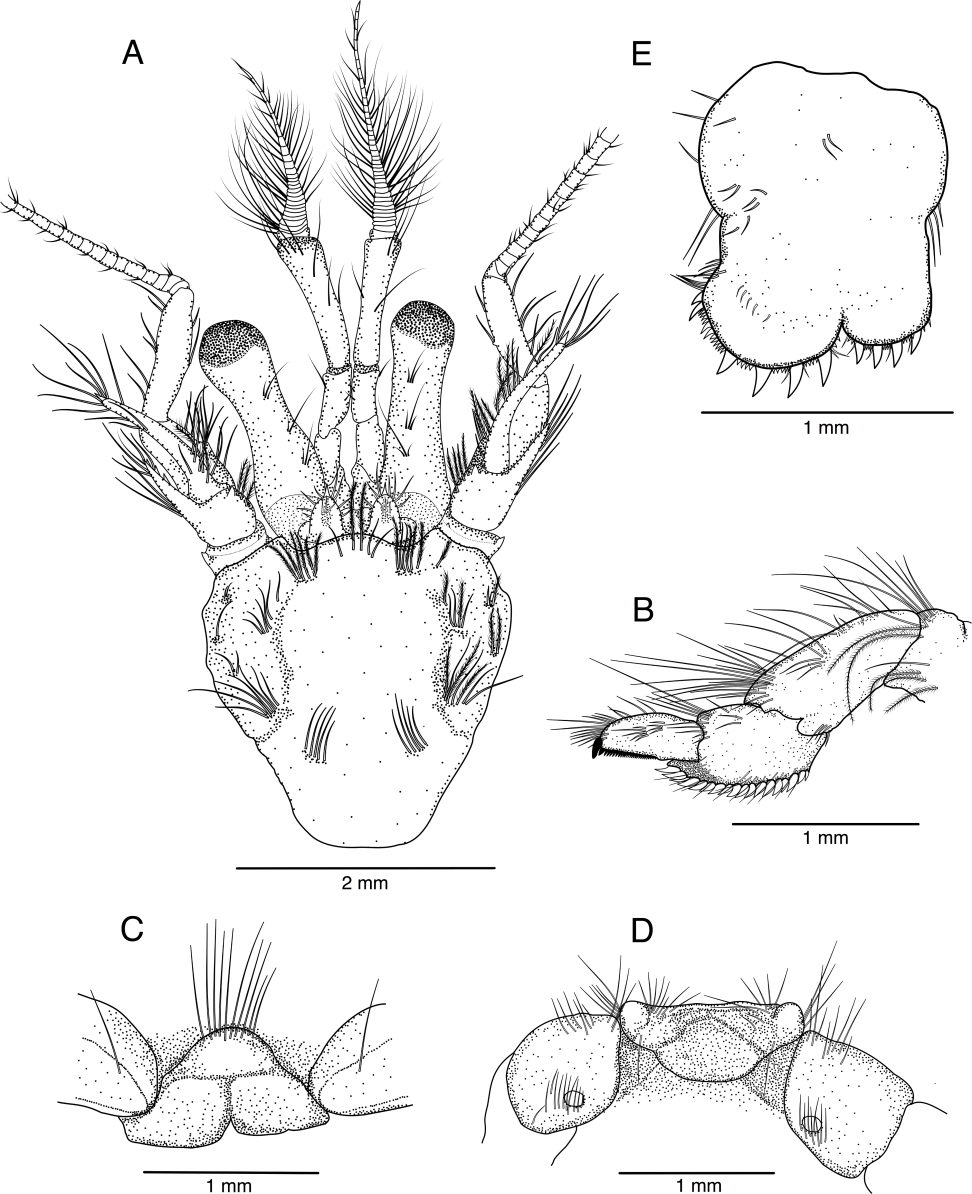
*Pagurus fraserorum* n. sp.. (A) ovigerous female paratype 2.4 mm (SAMC MB-A066770), shield and cephalic appendages, dorsal view; (B-E) male holotype 2.7 mm (SAMC MB-A066790), (B) dactylus, propodus, carpus and merus (in part) of fourth left pereopod, lateral view, (C) thoracic sternite 6 (third pereopods), ventral view, (D) coxae of fifths pereopod and thoracic sternite 8, ventral view, (E) telson, dorsal view.

**Fig 2:**
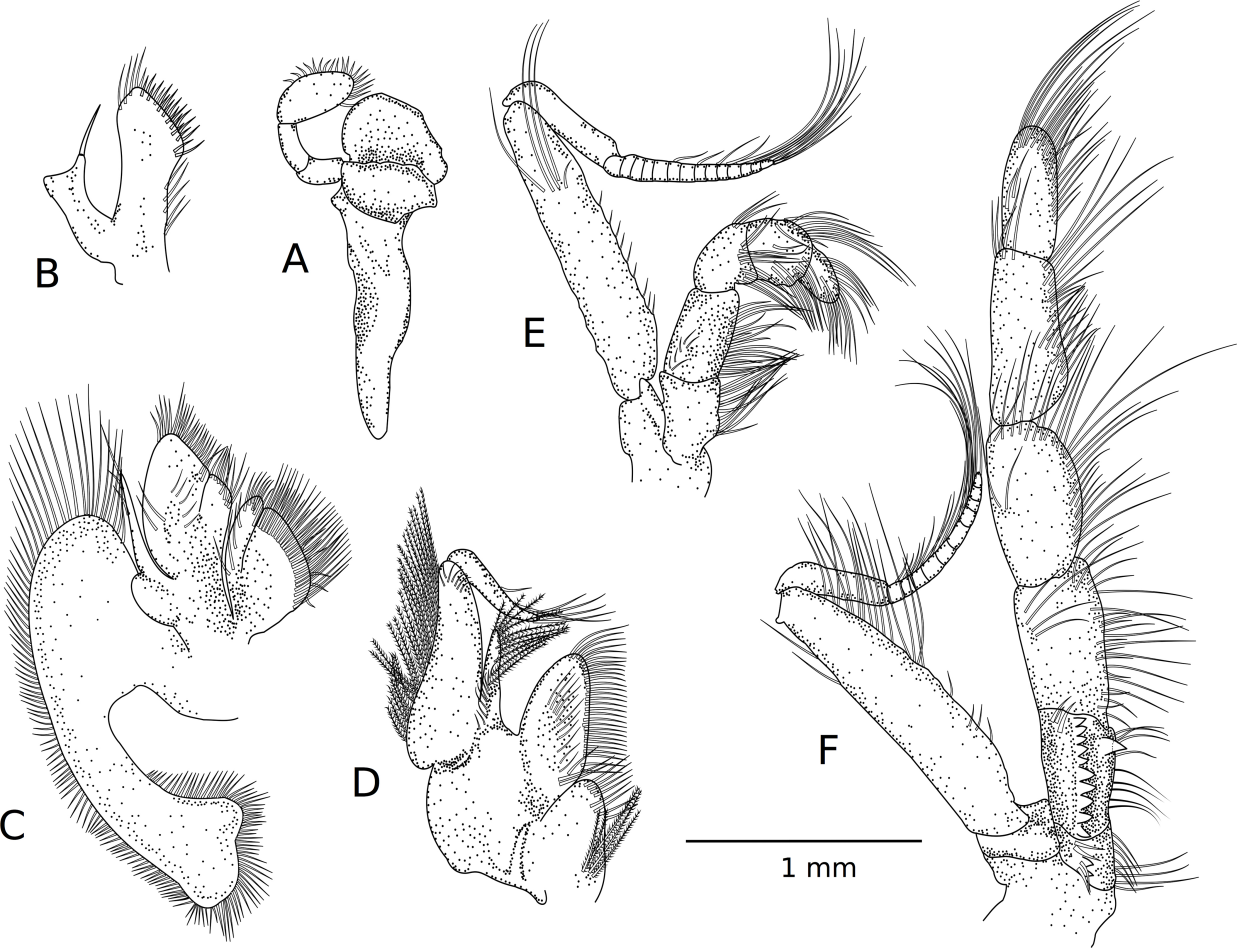
*Pagurus fraserorum* n. sp., drawings of left mouthparts (internal view) female paratype 2.4 mm (SAMC MB-A066407). (A) Mandible; (B) maxillule; (C) maxilla; (D) first maxilliped; (E) second maxilliped; (H) third maxilliped.

**Fig 3:**
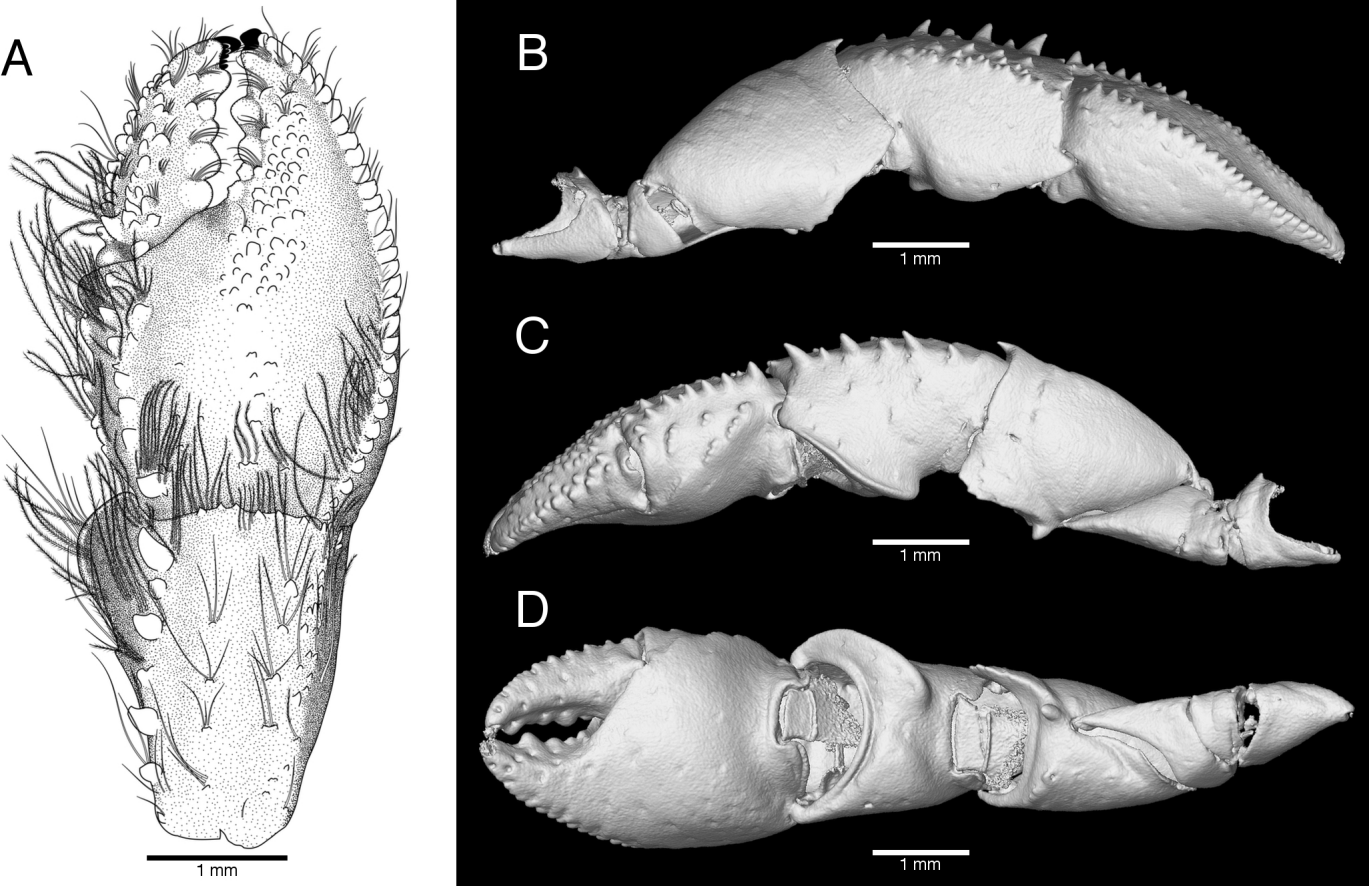
*Pagurus fraserorum* n. sp., drawing and microCT images of right cheliped of male holotype 2.7 mm (SAMC MB-A066790). (A) Drawing of chela and carpus, dorsal view; (B) microCT image of right cheliped, lateral view; (C) same, mesial view; (D) same, ventral view.

**Fig 4:**
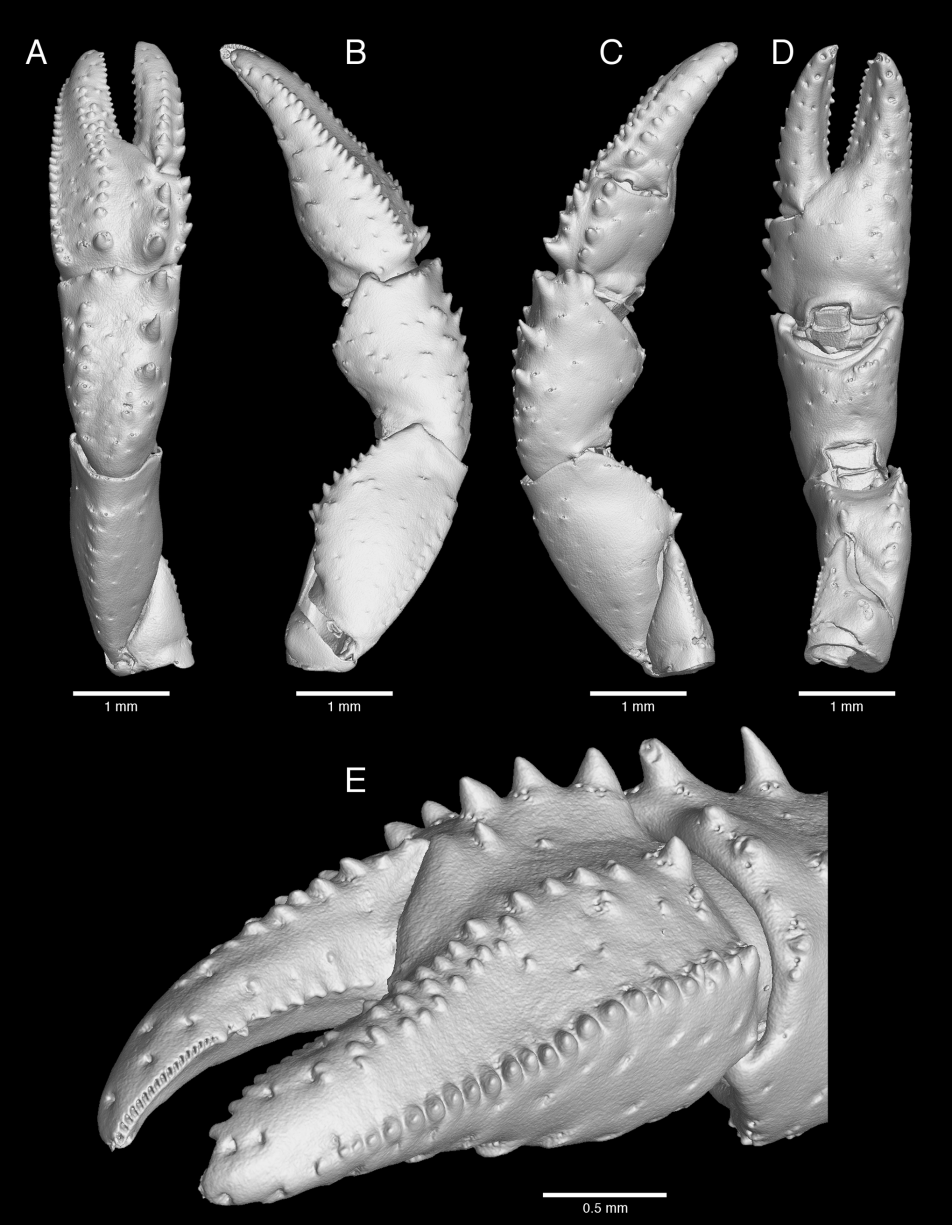
*Pagurus fraserorum* n. sp., microCT images of left cheliped of male holotype (SAMC MB-A066790). (A) dorsal view; (B) lateral view; (C) mesial view; (D) ventral view; (E) chela and part of carpus, laterofrontal view.

**Fig 5:**
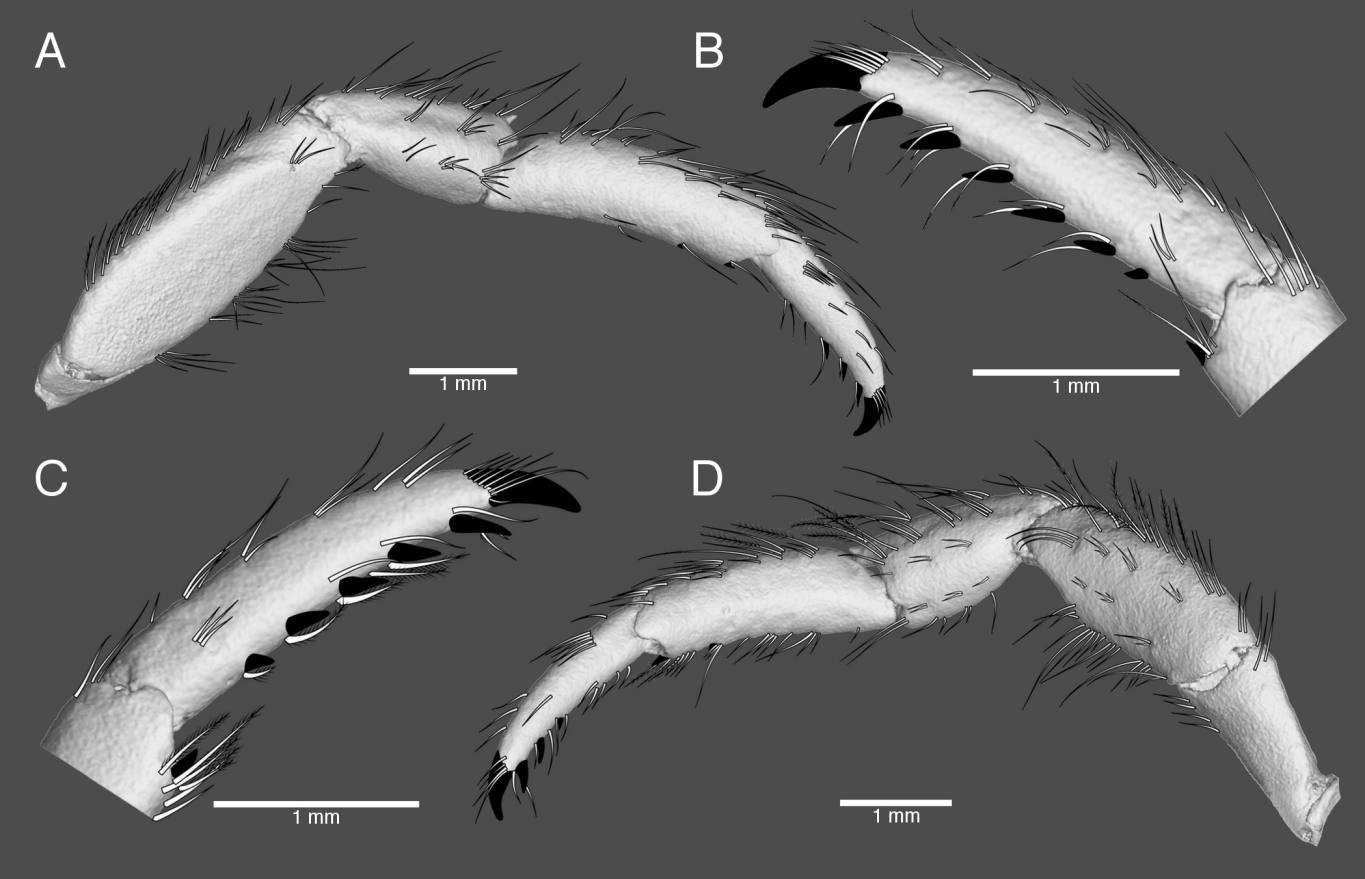
*Pagurus fraserorum* n. sp., microCT images with manually drawn setation and spinules and claw of pereopods of male holotype (SAMC MB-A066790). (A) second right, lateral view; (B) same, dactylus and distal part of propodus of second right, mesial view; (C) third left, dactylus and distal part of propodus, mesial view; (D) same, lateral view, drawing.

**Fig 6:**
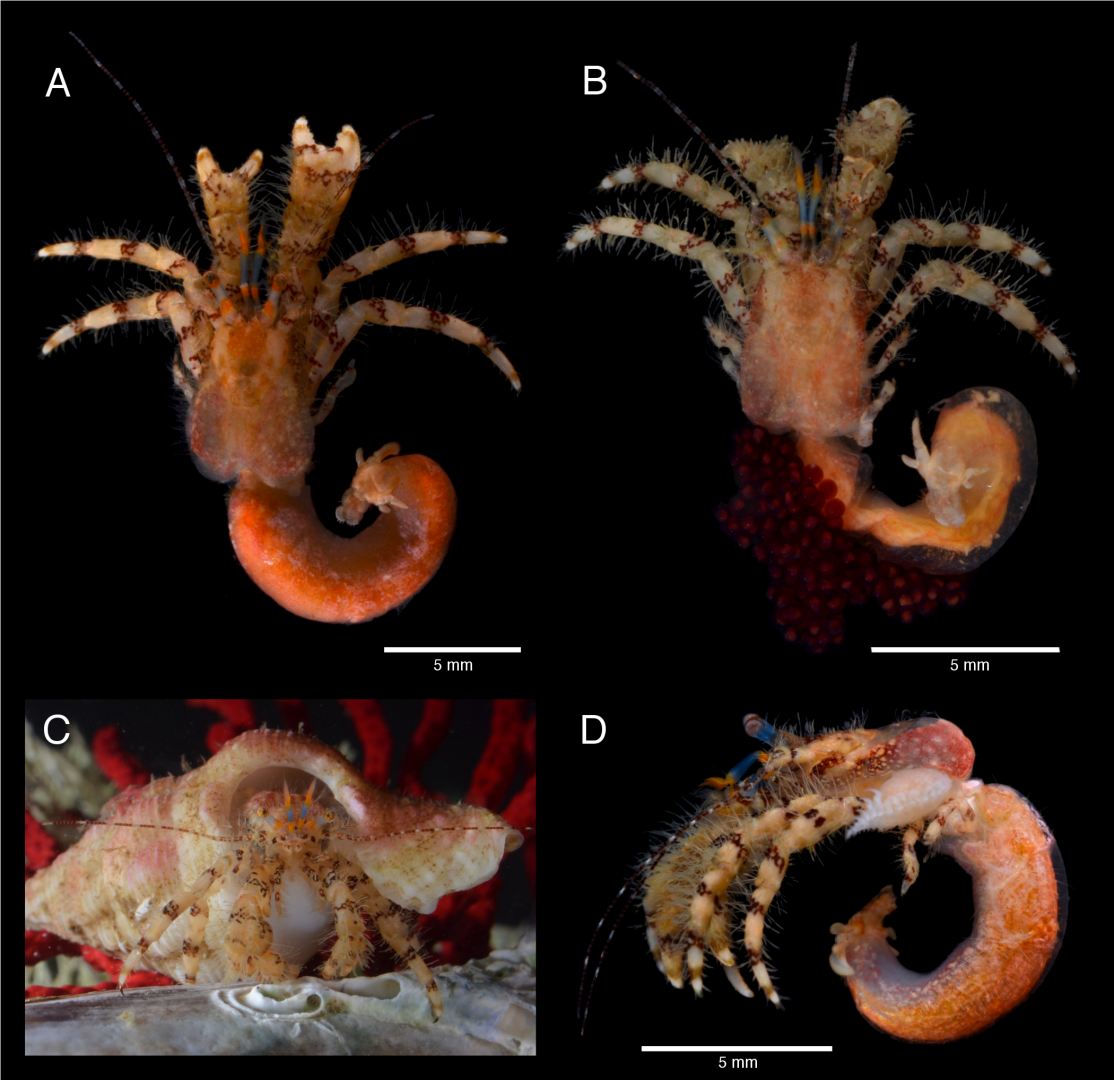
*Pagurus fraserorum* n. sp., colouration in life or fresh. (A) male holotype 2.7 mm (SAMC MB-A066790), dorsal view; (B) ovigerous female paratype 2.4 mm (SAMC MB-A066770), dorsal view; (C) holotype, frontal view, in shell; (D) female paratype 2.4 mm (SAMC MB-A066407), left lateral view, parasitized by undescribed parasitic gills isopod *Pseudionella* sp..

Figs 1–6, S1 Fig, S1–S3 Videos

#### Type material

Holotype, from KwaZulu-Natal, South Africa. 15 Oct 2015, off Hibberdene, S 30° 34.92’, E 30° 34.86’, reef, 20 m deep, SCUBA, 1 male 2.7 mm (SAMC MB-A066790).

Paratypes, all from KwaZulu-Natal, South Africa. 14 Oct 2015, off Pumula, S 30° 38.34’, E 30° 32.94’, reef, 20 m deep, SCUBA, 1 female 2.4 mm (SAMC MB-A066407), 1 juv. male 1.4 mm (SAMC MB-A066408), 1 ovig. female 1.2 mm (SAMC MB-A066409), 1 male 2.1 mm (SAMC MB-A066410), 1 female 2.0 mm (SAMC MB-A066411); 15 Oct 2015, off Hibberdene, S 30° 34.92’, E 30° 34.86’, reef, 20 m deep, SCUBA, 1 ovig. female 2.4 mm (SAMC MB-A066770), 1 male 1.9 mm, 1 female 2.3 mm (CBM-ZC 14133).

Other material. One specimen not collected, Aug 2015, Vetch’s Pier, Durban, KwaZulu-Natal, S 29° 52.02’, E 31° 03.00’, 6 m, SCUBA, photographed *in situ* by Georgina Jones.

#### Description

Eleven pairs of biserial gills.

Carapace flattened dorsoventrally. Shield (S1 Fig and Fig 1A) about as broad as long; dorsal surface nearly flat except for sloping lateral parts, with scattered low, blister-like tubercles (only visible when stained with methylene blue) and rows of tufts of short, distally plumose setae along rather distinct paragastric grooves; anterior margin between rostrum and lateral projections shallowly concave; anterolateral margins sloping, very weakly concave or straight; posterior margin roundly truncate. Rostrum broadly rounded or very broadly triangular, not reaching (or just reaching) level of lateral projections; dorsal surface usually with few short paired setae. Lateral projections roundly triangular, each armed with minute spine or unarmed. Posterior carapace (S1 Fig, S1 Video, in part) slightly shorter than shield and about half as long as wide, cardiac region flanked by sulci cardiobranchiales slightly calcified, with scattered low, blister-like tubercles; branchiostegite to branchial regions membranous, with sparse tufts of short to moderately-long plumose setae.

Ocular peduncles (including cornea) (S1 Fig and Fig 1A) about 0.75 length of shield, weakly inflated in proximal half; cornea weakly dilated, its width about 0.25 length of peduncular length; dorsal surface mesially with 3 or 4 tufts of setae, ventromesial surface also with 1 or 2 individual setae, or tufts of few setae. Ocular acicles suboval, separated basally by 0.5 width of 1 acicle, each with a small submarginal spine and some short setae on distal margin and dorsal surface; dorsal surface shallowly-concave. Interocular lobe fully exposed, visible in dorsal view, medially concave.

Antennular peduncles (S1 Fig and Fig 1A), when fully extended, overreaching distal corneal margins by 0.3–0.5 length of ultimate segment. Ultimate segment about twice as long as penultimate segment, widened distally, with sparse setae increasing in length distally on dorsal surface to dorsolateral distal angle. Penultimate segment with few short setae. Basal segment with setose, rounded laterodistal lobe; statocyst lobe with small distal spine; ventromesial distal angle unarmed.

Antennal peduncles (S1 Fig and Fig 1A), when fully extended, overreaching distal corneal margins by 0.5 length of fifth segment. Fifth and fourth segments with few short setae on lateral and mesial faces (mesial setae on fourth segment stiff). Third segment unarmed on ventromesial distal angle, but with tuft of short to long stiff setae. Second segment with dorsolateral distal angle produced, reaching to about mid-length of fourth segment, terminating in simple spine partially obscured by tuft of short to long, stiff setae, with few minute spinules on dorsomesial margin; dorsomesial distal angle with small spine; mesial margin with short to long setae. First segment with very small laterodistal spine; ventrodistal margin mesially with small process distally with excretory pore. Antennal acicle reaching to mid-length or distal margin of cornea, moderately arcuate, terminating in simple spine encircled and partially obscured by long stiff setae; dorsomesial margin with row or tufts of moderately-long to long, often distally plumose setae. Antennal flagellum more than 3 times length of shield; each article with some short setae on distal margin, length of setae 0.5–1.0 times length of 1 article.

Mouthparts as figured. Vast majority, but particularly mandible (Fig 2A) and second maxilliped (Fig 2E), not showing distinguishing features. Maxillule (Fig 2B) with external lobe of endopod moderately well developed, triangular, not recurved; internal lobe well produced, with 1 apical seta. Maxilla (Fig 2C) with endopod exceeding anterior margin of scaphognathite; endopod basally with small lobe. First maxilliped (Fig 2D) with endopod reaching level of distal third of exopod. Third maxilliped (Fig 2F) moderately stout, setation normal; dactyl about 0.5 length of propodus; carpus unarmed; merus also without conspicuous armature; ischium with crista dentata with about 10 corneous teeth increasing in size and more widely spaced proximally, and with 1 strong accessory tooth; basis with 2 minute denticles on mesial margin; exopod exceeding mid-length of carpus.

Chelipeds unequal and dissimilar, right appreciably stronger than left. Right cheliped (Fig 3A–3D) moderately stout, not particularly elongated, even in males. Chela subovate in dorsal view, about 1.4 times as long as wide. Dactyl subequal in length to palm (measured along mesial margin), not overlapped by fixed finger when closed; dorsal surface slightly elevated along midline, bearing 1–3 irregular longitudinal rows of tubercles and sparse tufts of short stiff setae, and also with longitudinal row of stiff setae along cutting edge; mesial surface with irregular double row of small tubercles decreasing in size distally and with tufts of stiff, simple or plumose setae becoming shorter distally; ventral surface nearly smooth, with 2 rows of tufts of stiff setae; cutting edge with row of 5 rounded or bluntly-triangular calcareous teeth in proximal 0.8 and short row of minute corneous teeth in distal 0.2, terminating in small corneous claw. Palm 0.75 times as long as carpus; dorsolateral margin slightly elevated, clearly delimited with row of small, rounded tubercles extending onto fixed finger (tubercles becoming smaller distally) and bearing tufts of long, thick plumose setae not extending onto fixed finger; dorsomesial margin also slightly elevated, delimited with double row of small to moderately small spines and tufts of long, densely plumose setae; dorsal surface with broad, blunt ridge extending from midlength of palm onto fixed finger, leading proximally to row of small, simple or multifid tubercles; surface of median ridge covered with small, low, rounded tubercles; spaces either side of median ridge very shallowly concave, with sparse minute tubercles; lateral surface nearly perpendicular, without conspicuous armature, with longitudinal row of rows or tufts of short to moderately-long plumose setae adjacent to dorsal margin; mesial face with several short, obliquely transverse ridges or elevations each bearing row or tuft of long setae (setae on dorsal side thickly plumose, those on ventral side generally simple); ventral surface slightly convex, smooth, with few tufts of simple setae. Cutting edge of fixed finger with 3–4 prominent calcareous teeth, terminating in small corneous claw. Carpus moderately widened distally, 1.1–1.2 length of merus; dorsal surface with very low, small, distally multidenticulate protuberances, each bearing short to long stiff plumose setae, arranged in 2 rows; dorsomesial margin clearly delimited with row of small spines increasing in size distally and becoming blunt and low proximally, and tufts of long stiff setae; dorsolateral margin at least distally armed by 1–3 irregular rows of acute small tubercles or tiny spines, and also with row of tufts of long, stiff setae decreasing in length proximally, in smaller specimens rounded and undelimited; dorsodistal margin with row of long, stiff, often plumose setae partially obscuring proximal part of palm; lateral surface nearly flat, perpendicular, smooth, with few short setae; mesial face slightly concave, with scattered tufts of short to long, simple setae, ventromesial margin sharply crested, somewhat flared, visible in dorsal aspect; ventral surface convex, with scattered tufts of short to moderately long setae. Merus subtriangular; dorsal surface almost smooth, with few short setae and low subdistal ridge extending onto lateral and mesial faces, dorsodistal margin with small spine partially obscured by adjacent stiff setae; lateral surface only with few short setae, ventrolateral distal margin minutely denticulate; mesial surface with scattered stiff setae distally, ventromesial margin crested with prominent tubercle proximally, ventromesial distal angle produced into subtriangular plate, its ventral margin minutely denticulate distally or less produced, rounded, bearing few minute tubercles; ventral surface with tiny, low tubercles and few tufts of setae, but without conspicuous tubercle or spine. Ischium unarmed. Coxa with few low, blister-like tubercles on ventral surface and tufts of setae on ventromesial distal angle.

Left cheliped (Fig 4A–4E) moderately long and slender, reaching to level of mid-length of dactyl of right cheliped; setae stiff, those of dorsal surface of palm particularly densely plumose. Chela elongate subovate in dorsal view, 1.9 times as long as wide (measured at greatest width at base of dactyl). Fingers leaving narrow hiatus in proximal third when closed. Dactyl gently curving ventrally, 1.6–1.9 times as long as palm; dorsal surface slightly elevated along midline, bearing row of tiny tubercles in proximal 0.4 and tufts of setae extending to tip (setae of proximal tufts longer, thickly plumose, those of distal tufts becoming shorter, thin and simple); dorsomesial margin not delimited; mesial face with row of tufts of setae along midline (setae of proximal 2 or 3 tufts long, plumose, those of distal tufts shorter and simple), bases of setal tufts (in particular those of 2 most proximal) forming small tubercles; ventral surface smooth, with tufts of short setae arranged in 2 rows; cutting edge with row of minute, calcareous tubercles in proximal half and row of minute, corneous teeth in distal half, terminating in strong corneous claw. Palm approximately half length of carpus; dorsolateral margin distinctly delimited with row of tiny tubercles extending onto midlength to proximal 0.3 of fixed finger and tufts of long, plumose setae; dorsomesial margin rounded, but with row of 4 small spines or tubercles; dorsal surface medially with tiny tubercles arranged in irregular 2 rows and extending from proximal part of palm to midlength of fixed finger and prominent tufts of long plumose setae, and with longitudinal row of blunt tubercles and prominent tufts of long, plumose setae mesial to midline; lateral surface nearly perpendicular, smooth, with few tufts of short setae; mesial surface smooth, with few tufts of setae; ventral surface slightly convex, with few tiny tubercles laterally at base of fixed finger, and with scattered tufts of long setae; fixed finger with cutting edge with row of tiny, triangular calcareous teeth, terminating in strong, spooned corneous claw; ventral surface with 2 rows of tufts of setae. Carpus subequal in length to merus; dorsal surface slightly depressed or nearly flat, smooth or with very small tubercles, with several short, simple and long, plumose setae; dorsomesial margin with 3 small spines increasing in size distally, and sometimes followed by 2 or 3 tiny tubercles or low protuberances proximally, all accompanied with tufts of simple or plumose, long setae; dorsolateral margin unarmed or with tubercles or small spines increasing in size distally, all spines or tubercles bearing tufts of short to long setae; lateral surface with few low protuberances and tufts of setae dorsally, otherwise smooth; mesial surface with scattered tufts of long setae, distomesial angle triangularly produced; ventral surface strongly convex, with scattered tufts of short to long setae. Merus with row of sparse setae on dorsal margin and with short transverse ridge subdistally, dorsodistal margin unarmed; lateral surface almost smooth, only with few setae, ventrolateral margin with few minute denticles; mesial surface also smooth, with few setae, ventromesial margin unarmed distally, proximally with blunt tubercle; ventral surface with scattered tuft of short to long setae, but without conspicuous spines or tubercles. Ischium with ventromesial margin faintly denticulate; surfaces with scattered tufts of short, thin setae. Coxa similar to that of right cheliped.

Ambulatory legs (Fig 5A–5D) similar from left to right, moderately stout, with tufts of long, stiff or slightly-shorter, plumose setae predominantly along dorsal and ventral margins, right second pereopod slightly overreaching tip of extended right cheliped. Dactyli approximately as long as propodi and about 5 times longer than broad, in dorsal view straight, in lateral view gently curving ventrally, terminating in moderately long (0.15–0.25 of entire length of each dactylus), curved corneous claw; dorsal surfaces each with sparse row of tufts of setae; lateral and mesial faces sparsely setose, latter without corneous spinules; ventral margins each with row of 5 or 6 (rarely 4) corneous spines, increasing in size and becoming more widely spaced distally, and row of tufts of plumose setae (setae most prominent in left third pereopod). Propodi slightly curved ventrally, 1.2–1.4 length of carpi; dorsal surfaces each with single or double row of tufts of long setae; lateral and mesial faces smooth, only with few very short setae; ventral surfaces each with 1–3 corneous spinules, including one at distal margin and row of tufts of short to long setae (distal setae plumose). Carpi each with dorsodistal spine; dorsal surface with row of tufts of long setae; lateral surface also with 2 rows of tufts of short to long, plumose setae with minute setules; ventral surface with few tufts or individual setae. Meri each without conspicuous spines or tubercles (2 right usually with minute denticle on ventrolateral distal angle); dorsal and ventral margins each with row of long, plumose setae (with very short setules); lateral faces smooth, with only few short setae. Ischia with few tufts of short to long setae on dorsal and ventral margins; without conspicuous spines or tubercles. Females with paired gonopores on coxae of third pereopods.

Fourth pereopods (Fig 1B) semichelate, subequal in length from left to right, with subterminal tuft of short setae on dorsal margin of dactyli, stiff long setae and tufts of setae on dorsal margin of propodi to meri and ventromesial margin of meri. Dactyli nearly straight, with convex dorsal margin, terminating in small, strongly-curved corneous claw; ventral margin with closely-set, microscopic corneous teeth; no preungual process. Propodal rasp consisting of a single row of corneous scales.

Fifth pereopods chelate, propodal rasp extending slightly beyond midlength of segment. Coxae of male (Fig 1D) each with gonopore partially masked by tuft of setae.

Sternite of somite XII (thoracomere 6, third pereopods)(Fig 1C) with subsemicircular, wide anterior lobe, bearing several setae on anterior surface. Sternite of somite XIV (thoracomere 8, fifth pereopods)(Fig 1D) wide, with row of short setae on anterior margin; anterolateral lobes widely separated, slightly produced.

Pleon dextrally twisted. Male with 3 (third to fifth) unpaired, unequally biramous left pleopods. Female with slightly unequally biramous second to third pleopods; fourth pleopod unequally biramous with reduced endopod; fifth pleopod with endopod greatly reduced: exopod well-developed.

Uropods and telson asymmetrical. Telson (S1 Fig, in part, Fig 1E) with distinct lateral indentations separating anterior and posterior lobes; posterior lobes rounded to subrectangular, unequal, with deep V-shaped median cleft; right posterior lobe more rounded and about twice as large as left one; terminal margins each with 4–6 (left usually with 4) prominent spines interspersed by minute spines and very short setae; lateral margins with rows of stiff setae.

#### Coloration (in life or fresh; Fig 6A–6D)

Shield pink to maroon and mottled cream to white, usually darker anteriorly. Posterior portion of carapace pink mottled cream, branchiostegites usually dark brown and mottled cream. Ocular peduncle sky-blue with 2 bright orange transverse bands, one distally just proximal to base of cornea not extending to ventral surface, and one in second quarter from proximal; cornea yellow golden with very small red dots. Antennular peduncle with penultimate segment sky-blue in proximal and bright orange in distal half, ultimate segment azure and with bright orange band distally; flagella bright orange. Antennal peduncle cream with maroon stripes and bands, fifth segment with lateral and mesial longitudinal maroon stripe accompanied by small, white spots; flagellum in proximal half laterally and mesially with intermitted longitudinal maroon stripes leading distally into broadly transverse bands. Chelipeds similar in coloration, cream with maroon bands mottled cream; dactyl and fixed finger with weak pale brown band in distal half and maroon band or patch in proximal half, palm with irregular maroon patterns; carpus and merus proximally with transverse band of irregular maroon pattern. Ambulatory legs similar to chelipeds; dactyl with pale brown band in distal half and proximal transverse maroon band mottled cream; equally-spaced transverse maroon bands occurring along ambulatory legs, with 1 band each near middle of propodus, proximal portion of carpus, at midlength of merus, proximal portion of merus and distal half of ischium. Uropods and telson cream to mottled pink. Pleon orange to pink. Eggs deep maroon.

#### Distribution

Known from rocky reefs off Pumula and Hibberdene, near Port Shepstone, KwaZulu-Natal, South Africa, and from a photographic record from Vetch’s Pier, Durban, South Africa; 6–20 m depth.

#### Parasites

Host of an undescribed parasitic bopyrid isopod *Pseudionella* sp. (Fig 6D) that is currently being described by Williams & Landschoff, and of a second parasitic isopod, *Pseudione* sp., both inhabiting the branchial chamber.

#### Etymology

The species is named after the Fraser family, in particular Valda and Mike, but also to their son Allan and honours their deep passion for the marine world and the many exciting species discoveries they have made in South African waters. Mike and Valda generously hosted the senior author and his supervisor Charles L. Griffiths over the collection trip in October 2015, when this species was first recorded. This sampling would have not been possible without the use of the facilities and guidance they provided.

#### Taxonomic remarks

The new species is clearly not referable to any of the known South African species presently referred to *Pagurus*, but appears close to *P*. *boriaustraliensis* from northern and northwestern Australia, and *P*. *pitagsaleei* from Thailand and Taiwan. The taxonomy of *P*. *boriaustraliensis* and *P*. *pitagsaleei* has to be re-evaluated, as the latter might be a synonym of the former (see genetics results and discussion), but in the in the following comparison they are considered separate species. *Pagurus fraserorum* n. sp., *P*. *boriaustraliensis* and *P*. *pitagsaleei* all share strong similarities in the shape and ornamentation of the shield and cephalic appendages, characteristically ridged palms of the chelipeds, sharply crested, flared ventromesial margins of the carpus and merus of the right cheliped at least in males, and general shape of the eighth thoracic sternite and telson. However, morphological characters that separate *P*. *fraserorum* n. sp. from the other two species are the shorter ocular peduncles, which are exceeded by approximately half the length of the ultimate article of the antennular peduncle (only slightly exceeded in *P*. *boriaustraliensis* and *P*. *pitagsaleei*), the meri of the chelipeds that lack distinct tubercles on the proximal ventral surface (distinct tubercle(s) occur in both the other species), and the unarmed ventral margin of the merus of the right second pereopod (armed with one or more spines in *P*. *boriaustraliensis*, and with one spine in *P*. *pitagsaleei*). Furthermore, in *P*. *pitagsaleei* the dorsolateral margin of the carpus of the right cheliped is sharply delimited by a row of prominent spines [48,57,58], in *P*. *boriaustraliensis* it is less strongly delimited, but bears considerably large spines [47]. In *P*. *fraserorum* n. sp. the dorsolateral margin of the carpus of the right cheliped is armed with 1–3 irregular rows of acute tubercles or tiny spines. Furthermore, a strong asymmetry in the dimensions of the fourth pereopods, present in *P*. *pitagsaleei* [57], is absent in the new species.

In addition to morphological characters, living colouration is useful to differentiate these species [47,48,57,58]. The ocular peduncles of *P*. *boriaustraliensis* and *P*. *pitagsaleei* were reported as cream and gray-white respectively, and have longitudinal stripes, whereas in *P*. *fraserorum* n. sp. they are sky blue with two bright orange transverse bands, and without longitudinal stripes. The antennular peduncles are described as generally cream (the penultimate segment with dark stripes, and the flagellum bright orange) in *P*. *boriaustraliensis* and generally grayish-white (with dark red stripes and patches, ultimate segment orange distally) in *P*. *pitagsaleei*, whereas in the new species they are sky-blue to azure with bright orange bands and bright orange flagellum, but not with longitudinal stripes or dark patches. Similar differences can be seen in the colours of chelipeds and ambulatory legs. While the chelipeds and pereopods in *P*. *boriaustraliensis* are cream-brown with dark brown lines and in *P*. *pitagsaleei* grayish-white and black with short black or reddish-black stripes, in the new species they are cream with maroon bands mottled cream, and do not have longitudinal stripes.

In South Africa, *P*. *fraserorum* n. sp. is easily distinguishable from all other local species presently referred of *Pagurus* by the characteristic shape, armature, and setation of the chelipeds, pereopods, and telson. Among South African representatives of the genus, *P*. *fraserorum* n. sp. is perhaps morphologically most similar to *P*. *liochele*, with which it shares general characters of the right cheliped, such as some delimitations of the dorsolateral and dorsomesial margins, as well as the wing-like projection of the ventromesial face of the merus and carpus. However, for example, *P*. *fraserorum* n. sp. differs from *P*. *liochele* by having a characteristic median ridge on the palm of the right cheliped, the less evenly distributed, less rounded and slightly more acute tubercles on the dorsal surface of the right palm, and by having long and densely plumose setae in certain places of the chelipeds (both chelipeds in *P*. *liochele* are at most covered with short setae). In life, the two species can readily be identified by the colour, as *P*. *fraserorum* n. sp. has maroon bands around the chelipeds and pereopods, and *P*. *liochele* has longitudinal orange stripes on the dactyli and a sienna band on the propodi proximally [59].

#### Key to known South African species of *Pagurus*, adapted after McLaughlin & Forest (1999)

**1 a.** Dorsal surface of palm of chelipeds armed with spines………………………2
**1 b.** Dorsal surface of palm of chelipeds unarmed, or armed only, or mostly, with granules or small tubercles……………………………………….…………………………4
**2 a.** Right second pereopod with dorsal row of spines on propodus…………3
**2 b.** Right second pereopod without dorsal row of spines on propodus……………………………………………………… *Pagurus* sp. (see McLaughlin & Forest [35])
**3 a.** Ocular peduncles short, stout. Dorsal surface of palm of right chela with small tubercles or spines, strongest mesially and laterally; carpi of chelipeds each, or at least right, usually with prominent foramen on ventral surface ……………………………………*Pagurus carvicarpus* (Paul’son, 1785) [43]
**3 b.** Ocular peduncles moderately long and slender. Dorsal surface of palm of right chela with rows of spines, strongest in midline; carpi of chelipeds each, or at least right, without prominent foramen on ventral surface……………………………………………… *Pagurus cuanensis* Bell, 1845 [44]
**4 a.** Dactyls of ambulatory legs shorter to only slightly longer than propodi … …………………………………………………………………………………………………………5
**4 b.** Dactyls of ambulatory legs at least 1.5 length of propodi ………………………………………………………… *Pagurus prideaux* Leach, 1815 [46]
**5 a.** Ventromesial face of merus and carpus of right cheliped developed as wing-like projection in both sexes (Fig 3A and Fig 3C and 3D) ……………………………………………………………………………………………………6
**5 b.** Ventromesial face of merus and carpus of right cheliped not developed as wing-like projection in either sex ………………………………………………………………………………………… *Pagurus emmersoni* McLaughlin & Forest, 1999 [35]
**6 a.** Dorsal surface of right palm and merus evenly covered by blunt tubercles, surface of palm convex, not forming a longitudinal elevation in distal half……………………………………………*Pagurus liochele* (Barnard, 1947) [45]
**6 b.** Dorsal surface of right palm and merus medially unevenly covered with majorly blunt tubercles, surface of palm mesially and laterally concave, medially forming a longitudinal elevation in distal half extending onto fixed finger (Fig 3A) ………………………… *Pagurus fraserorum* n. sp. (S1 Fig, Figs 1–6, S1 Fig, S1 and S2 Videos)

#### 3D type data

The 3D data of the holotypic and one paratypic specimens derived from μCT scans are presented as interactive PDF (S1 Fig), still images (Figs 3B–3D and 4) overlaid with manually drawn morphological characters such as the setation or corneous spinules (Fig 5), as well as video clips viewable in the supplement section of this publication (S1–S3 Videos). Data processing as described in the materials section was used to remove noise and unwanted features, while maintaining the original data as reference. Because this cleaning process has advantages (revealing a nice and clean surface, illustrating calcified, well-defined taxonomic characters) and disadvantages (removing important information such as minute spines, corneous spinules, and setation), for presentation purposes it was applied to the holotype, but not to the female paratype. The scan-visualizations of the combined holotype in an interactive image (S1 Fig) allow the viewer to move, rotate and zoom into characters of interest. This interactive 3D image is limited to isosurface views and is reduced to information on relative grey values, e.g. lowering the possible quality of the image especially for fine features. It shows the calcified parts of the holotype as a whole and offers a rapid 3D overview of the specimen that includes the real size dimensions, such as relationships of *in situ* taxonomic characters from any possible angle. Its resolution is not sufficient for the study of finer structures, such as small spines or setae. If more detailed information is needed, the suite of other illustrations can be consulted. The 3D still images of the right cheliped (Fig 3B–3D) complement the line drawing of the dorsal view of the dactylus, propodus and carpus of the right cheliped (Fig 3A). Although the setation is not visualized in these images, the scan reconstructions provide 3D details in lateral, mesial and ventral view that better display the surface details of the drawing in accuracy. Being scanned separately, the high resolution scan of the left cheliped made it possible to show the minute cavities from which each setae arises (Fig 4). While this does not provide information about the setae itself (setation is well described in the description section), it precisely reveals the location of each seta on the cheliped. The 3D images of the pereopods (Fig 5) are surface renderings overlaid with manual drawings of the setation, the corneous spinules and of the claw. In addition, three movie clips are provided if more 3D views are needed. S1 Video shows the female paratype in a μCT reconstruction in which the ‘noise’ was not removed. Being wrapped and scanned in parafilm it includes scanning artefacts, but nevertheless a majority of setation, parts of the pleon and the eggs carried on the pleopods are all visible. S1 and S2 Videos are rotation movies of isosurface rendered separations of the right and left chelipeds. They resemble the scan images in Fig 3 and Fig 4. Because in Paguridae the chelipeds have significant identification characters, through the rotation movies the viewer can gain even more insights into the armature and appearance. All scan raw data are publicly available for download from the GigaScience Database (GigaDB) online depository [60], and more detailed information on scanning parameters and data quality can be retrieved from the accompanying datanote [50].

### Genetics

#### Material with COI barcodes

*Pagurus fraserorum* n. sp., off Pumula, KwaZulu-Natal, South Africa, 14 Oct 2015, S 30° 38.34’, E 30° 32.94’, 20 m reef, female 2.4 mm, BOLD: SEAKY SEAKY1145-16, GenBank: MH482096 (paragenetype) (SAMC MB066407), male 1.4 mm, BOLD: SEAKY1146-16, GenBank: MF695076 (paragenetype) (SAMC MB-A066408), ovig. female 1.2 mm, BOLD: SEAKY1147-16, GenBank: MF695072 (paragenetype) (SAMC MB-A066409), male 2.1 mm, BOLD: SEAKY1148-16, GenBank: MF695073 (paragenetype) (SAMC MB-A066410), female 2.0 mm, BOLD: SEAKY1149-16, GenBank: MF695071 (paragenetype) (SAMC MB-A066411). *Pagurus boriaustraliensis*, Dampier Archipelago, Western Australia, 30 Aug 1999, S 20° 29.70’, E 116° 35.88’, 1 male 4.1 mm, BOLD: GMDEC006-18, GenBank: MH670467 (WAM C27981), 8 Sep 1999, S 20° 27.48’, E 116° 39.54’, 5 m, 1 male 4.5 mm, BOLD: GMDEC007-18, GenBank: MH670463 (WAM C28021); Long Reef, Western Australia, 12 Oct 2010, S 13° 57.18’, E 125° 46.56’, no depth, female 4.2 mm, BOLD: GMDEC004-18, GenBank: MH670461 (WAM C47033-1), 19 Oct 2010, S 13° 55.32’, E 125° 43.98’, no depth, 1 male 3.4 mm, BOLD: GMDEC008-18, GenBank: MH670468 (WAM C47468), 24 Oct 2010, S 13° 57.42’, E 125° 43.08’, no depth, 1 male 3.6 mm, BOLD: GMDEC009-18, GenBank: MH670462 (WAM C47469). *Pagurus* sp., Long Reef, Western Australia, 12 Oct 2010, S 13° 57.18’, E 125° 46.56’, female 2.9 mm, BOLD: GMDEC005-18, GenBank: MH670464 (WAM C47033-2). *Pagurus cuanensis*, Roman Rock, False Bay, South Africa, 13 May 2015, S 34° 10.80’, E 18° 27.48’, 20 m, ovig. female 6.6 mm, BOLD: SEAKY931-15, GenBank: MF695069 (SAMC MB-A066192). *Pagurus emmersoni*, off Pumula, KwaZulu-Natal, South Africa, 13 Oct 2015, S 30° 38.34’, E 30° 32.94’, 20 m reef, male 4.6 mm, BOLD: SEAKY1139-16, GenBank: MF695070 (SAMC MB-A066401). *Pagurus liochele*, Algoa Bay, Port Elizabeth, South Africa, 30 Apr 2015, S 33° 50.10’, E 25° 57.00’, 50 m, ovig. female 5.7 mm, BOLD: SEAKY878-15, GenBank: MF695074 (SAMC MB-A066845), male 7.1 mm, BOLD: SEAKY879-15, GenBank: MF695075 (SAMC MB-A066846); Buffels Bay, South Africa, 20 Jul 2015, 34° 05.40’, E 22° 58.68’, intertidal, male 5.5 mm, BOLD: SEAKY863-15, GenBank: MF695071 (SAMC MB-A066246). *Pagurus pitagsaleei*, Haikou, Kending, Pingtung County, Taiwan, 8 September 2005, 1 male 4.1 mm, BOLD: GMDEC011-18, GenBank: MH670466, 1 ovigerous female 4.3 mm, BOLD: GMDEC010-18, GenBank: MH670465 (NTOU A01441).

#### Comparison of COI barcodes

**Fig 7:**
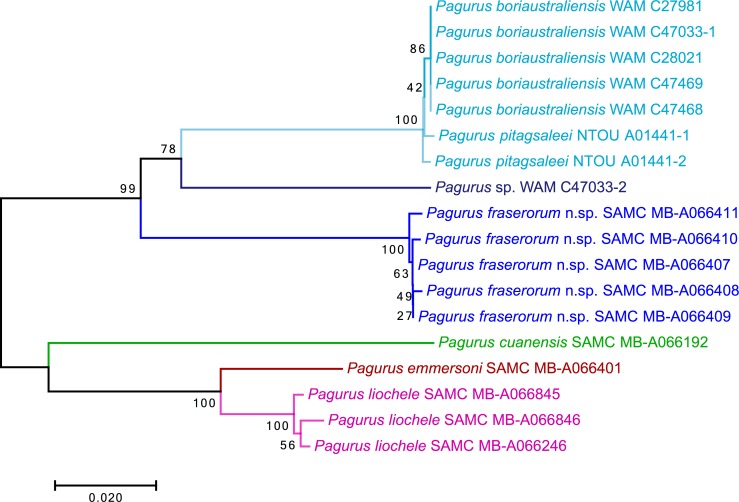
Neighbour-Joining (NJ) tree based on Kimura 2-parameter (K2P) distances [56], showing relationships among mitochondrial cytochrome *c* oxidase subunit I (COI) barcoding sequences of *Pagurus fraserorum* n. sp. and other related species of *Pagurus*. Nodal support from 1000 bootstrap replicates indicated above the branches.

In the NJ tree, barcodes of the five specimens of *P*. *fraserorum* n. sp. formed a monophyletic unit with 100% bootstrap support (Fig 7). Specimens identified as *Pagurus boriaustraliensis* from northwestern Australia and those of *P*. *pitagsaleei* from Taiwan were recovered to be genetically conspecific, forming another monophyletic unit, sister to the single sequence of an unidentified specimen of *Pagurus* sp. from northwestern Australia. Together these sister taxa formed a well-supported clade with *P*. *fraserorum* n. sp. with 99% bootstrap support. A second clade comprised *P*. *emmersoni* and *P*. *liochele*, well-supported (100%) as sister species, and *P*. *cuanensis* more divergent. Pairwise comparisons revealed that *P*. *liochele* and *P*. *emmersoni* were genetically much closer (0.039–0.044) to each other than to the morphologically very similar *P*. *fraserorum* n. sp. and *P*. *boriaustraliensis*/*P*. *pitagsaleei* (0.110–0.114, Table 1). The divergence between the conspecific specimens identified as *P*. *boriaustraliensis* or *P*. *pitagsaleei* was low (0.003). The unidentified *Pagurus* sp. was slightly more distant to *P*. *fraserorum* n. sp. (0.115–0.117) than to *P*. *boriaustraliensis*/*P*. *pitagsaleei* (0.096–0.100).

**Table 1:** Pairwise Kimura 2-parameter (K2P) distances [56] among mitochondrial cytochrome *c* oxidase subunit I (COI) barcode sequences of *Pagurus fraserorum* n. sp. and other related species of *Pagurus*; values highlighted for comparisons of *P*. *fraserorum* with *P*. *pitagsaleei*/*P*. *boriaustraliensis* and *Pagurus* sp., as well as for *P*. *liochele* with *P*. *emmersoni*.

		1	2	3	4	5	6	7	8	9	10	11	12	13	14	15	16	17
**1**	**P. fraserorum SAMC MB-A066407**																	
**2**	**P. fraserorum SAMC MB-A066408**	0.002																
**3**	**P. fraserorum SAMC MB-A066409**	0.000	0.002															
**4**	**P. fraserorum SAMC MB-A066410**	0.002	0.003	0.002														
**5**	**P. fraserorum SAMC MB-A066411**	0.003	0.005	0.003	0.005													
**6**	**P. boriaustraliensis WAM C47468**	**0.110**	**0.112**	**0.110**	**0.112**	**0.112**												
**7**	**P. boriaustraliensis WAM C47469**	**0.110**	**0.112**	**0.110**	**0.112**	**0.112**	0.000											
**8**	**P. boriaustraliensis WAM C28021**	**0.110**	**0.112**	**0.110**	**0.112**	**0.112**	0.000	0.000										
**9**	**P. boriaustraliensis WAM C27981**	**0.110**	**0.112**	**0.110**	**0.112**	**0.112**	0.000	0.000	0.000									
**10**	**P. boriaustraliensis WAM C47033-1**	**0.110**	**0.112**	**0.110**	**0.112**	**0.112**	0.000	0.000	0.000	0.000								
**11**	**P. pitagsaleei NTOU A01441-1**	**0.112**	**0.114**	**0.112**	**0.114**	**0.114**	0.003	0.003	0.003	0.003	0.003							
**12**	**P. pitagsaleei NTOU A01441-2**	**0.110**	**0.112**	**0.110**	**0.112**	**0.112**	0.003	0.003	0.003	0.003	0.003	0.003						
**13**	**Pagurus sp. WAM C47033-2**	**0.115**	**0.117**	**0.115**	**0.114**	**0.117**	0.098	0.098	0.098	0.098	0.098	0.100	0.098					
**14**	**P. liochele SAMC MB-A066846**	0.155	0.157	0.155	0.157	0.155	0.142	0.142	0.142	0.142	0.142	0.141	0.142	0.146				
**15**	**P. liochele SAMC MB-A066246**	0.149	0.151	0.149	0.151	0.149	0.140	0.140	0.140	0.140	0.140	0.139	0.141	0.144	0.006			
**16**	**P. liochele SAMC MB-A066845**	0.151	0.153	0.151	0.153	0.151	0.138	0.138	0.138	0.138	0.138	0.137	0.139	0.138	0.006	0.006		
**17**	**P. emmersoni SAM CMB-A066401**	0.153	0.155	0.153	0.155	0.157	0.151	0.151	0.151	0.151	0.151	0.150	0.152	0.142	**0.044**	**0.044**	**0.039**	
**18**	**P. cuanensis SAMC MB-A066192**	0.161	0.163	0.161	0.163	0.161	0.176	0.176	0.176	0.176	0.176	0.174	0.174	0.170	0.133	0.133	0.129	0.131

## Discussion

### The South African sibling

In South Africa, *Pagurus* is the only genus of hermit crabs which has been the subject of a relatively recent revision [35]. With the discovery of *Pagurus fraserorum* n. sp. in shallow waters on the southern coast of KwaZulu-Natal, there are now six (plus one undescribed) species reported in the region. Of these, *P*. *emmersoni*, *P*. *liochele*, and *P*. *fraserorum* n. sp. appear to be national endemics. The new species can be seen as the South African counterpart being sister to the species *P*. *boriaustraliensis/P*. *pitagsaleei*, which occur on the other side of the Indian Ocean. Our study has shown the strong character agreement and only slight morphological differences between the new species and *P*. *boriaustraliensis/P*. *pitagsaleei*, yet the available molecular data show that genetic species divergence can be reasonably high despite such morphological similarity (>10% *P*. *fraserorum* n. sp. to *P*. *boriaustraliensis/pitagsaleei*, Fig 7, Table 1). In comparison, the genetic difference between *P*. *emmersoni* and *P*. *liochele* (0.039–0.044) is small, yet these two South African species are easily distinguishable by both morphological characters and colouration [35]. Colouration has frequently been shown to be reliable and sufficient for the identification of many hermit crab species (e.g. see Results in [30–36], as natural selection presumably accelerates colour differentiation [61]. This is again evident from the considerable differences in colour patterns between *P*. *fraserorum* n. sp. and *P*. *boriaustraliensis*/*P*. *pitagsaleei*, species which show few distinguishing features in terms of their structural morphology. In fact, the distinctively coloured ocular acicles, antennae and ambulatory legs shown by *P*. *fraserorum* n. sp. were the characteristics which initially led us to suspect that this might be a new species. Only later did this lead to the detection of morphological differences with its relatives.

The finding that specimens of *P*. *pitagsaleei* from Taiwan and specimens of *P*. *boriaustraliensis* from northwestern Australia are genetically conspecific was a surprising result, indicating that reassessment of these two taxa is necessary, but this is beyond the scope of this paper. The preliminary genetic data point to the possibility that *P*. *pitagsaleei* is a synonym of *P*. *boriaustraliensis*. The single small specimen (female, 2.9 mm, WAM C47033-2), in one sample with a larger specimen of *P*. *boriaustraliensis* (female, 4.2 mm, WAM C47033-1) has unfortunately lost its right cheliped. However, the dorsal surface of palm of the left cheliped is moderately convex and has a longitudinal row of spines instead of a medially elevated ridge. It is therefore neither *P*. *boriaustraliensis* nor *P*. *pitagsaleei*, but a closely related species. It is included here for comparison even if this small and damaged specimen could not be identified at this stage.

### Systematic account

The species-rich and heterogeneous genus *Pagurus* is systematically poorly understood [62], and requires revision [63,64]. The results of this study suggest a close relationship between *P*. *fraserorum* n. sp. and *P*. *boriaustraliensis/P*. *pitagsaleei*, although the taxonomy of the latter species pair has to be clarified. The two latter species have been discussed as potential allies of the *Pagurus anachoretus* group cf. Forest [65–67]. At present this group includes the following 12 species: *P*. *anachoretus* (Risso, 1827) [68] from the Mediterranean, *P*. *anachoretoides* Forest, 1966 [69], *P*. *gordonae* (Forest, 1956) [70], *P*. *laurentae* Forest, 1978 [65], *P*. *sourie* (Forest, 1952) [71], from the tropical eastern Atlantic, *P*. *liochele* (Barnard, 1947) [45], *P*. *emmersoni* Mclaughlin & Forest, 1999 [35] from South Africa, and *P*. *decimbranchiae* Komai & Osawa, 2001 [67], *P*. *fungiformis* Komai & Rahayu, 2004 [66], *P*. *hedleyi* (Grant & McCulloch, 1906) [72], *P*. *kulkarnii* Sankolli, 1962 [73], and *P*. *moluccensis* Haig & Ball, 1988 [74] from the Indo-West Pacific [35,65,66,75]. In addition, four more species from the tropical eastern Atlantic fit well into the general definition, but have a different cheliped form [35]. These species are *P*. *alcocki* (Balss, 1911) [76], *P*. *dartevellei* (Forest, 1958) [77], *P*. *fimbriatus* Forest, 1966 [69], and *P*. *triangularis* (Chevreux & Bouvier, 1892) [78]. Predominantly based on the presence of low, blister-like tubercles, which are darkly stained by methylene blue, Komai & Rahayu [66] assessed all the species formerly assigned to the *anachoretus* group, except for the two South African species *P*. *liochele* and *P*. *emmersoni*, as well as three species from the latter group (*P*. *dartevellei*, *P*. *fimbriatus*, and *P*. *triangularis*). Since all of the tested species carried such blister-like tubercles, and because these tubercles are currently not known from any other group [66], they (Komai & Rahayu) assumed that this character is useful to define a homogenous assemblage within the heterogeneous genus *Pagurus*. Strengthening this assumption, during the course of this study, the presence of tubercles was confirmed for both the additional South African species *P*. *liochele* and *P*. *emmersoni*. Therefore, of this group *P*. *alcocki* remains the last to be tested to determine whether it has this character.

Additionally, *P*. *fraserorum* n. sp., *P*. *boriaustraliensis* (tested for the Western Australian specimens in this study) and *P*. *pitagsaleei* all have blister-like tubercles predominantly on the shield that stain darkly in methylene blue. We also tested the small specimen *Pagurus* sp. for these blister-like tubercles, but this character should be better confirmed for larger specimens and when the identification and therefore placement of this species can be made based on more and better material. However, whether *P*. *fraserorum* and *P*. *boriaustraliensis*/*P*. *pitagsaleei* should be assigned to the *anachoretus* group remains questionable. Morgan [47] compared *P*. *boriaustraliensis* with *P*. *hedleyi* and *P*. *kulkarnii*, McLaughlin [48] discussed the affinities of all these three species with *P*. *pitagsaleei*, Komai & Osawa [67] suggested a close relationship between *P*. *boriaustraliensis*, *P*. *decimbranchiae*, *P*. *moluccensis* and *P*. *pitagsaleei* (as *P*. cf. *boriaustraliensis*), but none of the authors made a final determination of formal assignment. One of the main characteristics of the *anachoretus* group is the shape of the right cheliped, with the dorsal surface of the palm being more or less rounded [75]. Later, McLaughlin & Forest [35] added one supplemental character of the fourth pereopod consisting of a single row of scales, and Komai & Rahayu [66] added two supplemental characters of the presence of low, blister-like tubercles on the calcified integuments of the body and pereopods, as well as the widely separated anterolateral lobes on the eighth thoracic sternite. Moreover, Komai & Osawa [67] noted that members of the *anachoretus* group and their allies share the uniting feature of the terminal margins of the telson usually being armed with some spines interspersed by smaller spines or spinules. While *P*. *fraserorum* n. sp and *P*. *boriaustraliensis*/*P*. *pitagsaleei* agree with all these additional characters, they differ somewhat in the shape of the right cheliped. In particular, all three species share a remarkably similar right cheliped with the dorsal surface of the chela mesially and laterally concave, medially bearing a broadly elevated, tuberculated ridge extending onto the fixed finger, and also with tufts of long plumose setae.

Although analyses were confined to a single mitochondrial marker *P*. *fraserorum* n. sp., *P*. *boriaustraliensis*/*P*. *pitagsaleei* formed a well-supported clade, in which the small specimen of *Pagurus* sp. was nested, underpinning their close relationship (Fig 7). Furthermore, *P*. *liochele* and *P*. *emmersoni* clustered together with *P*. *cuanensis* (herewith confirmed to not have blister-like tubercles) as a second clade to the former. This might suggest that the group of *P*. *fraserorum* n. sp., *P*. *boriaustraliensis/P*. *pitagsaleei*, and potentially *Pagurus* sp., could indeed be homogeneous assemblage outside of the *anachoretus* group. However, these results are preliminary, as COI alone may not be sufficient in resolving phylogenetic relationships [79]. More extensive research and molecular work are needed to elucidate the phylogeny within this group of *Pagurus*.

### Three-dimensional visualizations

The present description of *P*. *fraserorum* n. sp. is the first description of a hermit crab to use a larger set of characters illustrated by μCT scan reconstructions. This includes an interactive PDF of the calcified structures of the holotype, a video of a female paratype as a whole, high-resolution images and videos of the holotypic chelipeds, as well as still images of 3D reconstructions of the pereopods overlaid by manually-drawn setation and corneous spinules. However, with the difficulties that emanate from soft tissue visualizations, at present, μCT scans of hermit crabs do not provide the full information of the physical material. These challenges are a reoccurring problem in tomography imaging of fine structures of invertebrates [2,4,9,80]. Because of the immediate trade-off between the size of the sample and the achievable resolution, a common method is to scan certain body parts separately using a narrower field of view, resulting in a higher possible resolution, as for example applied for studying setae in insects [81]. We used this same approach for the left cheliped of the holotype, yet the resolution of 5 μm voxel size remained insufficient for the accurate visualization of fine setae and corneous spines. We thus did not reach maximum resolutions of <1 μm as for example Hita Garcia et al. [81] did for ants, mainly because the marine hermit crabs required to be scanned wet. This meant that they had to be placed in a container or wrapped in parafilm during the scan and could not be placed as close to the x-ray source of the scanner. Also, being submerged or saturated in the preservation medium resulted in reduced image contrast, and test scans with stained specimens also did not lead to remarkably improved results. Our findings are therefore congruent with Faulwetter et al.’s [14] consideration that not all taxonomic characters can be adequately displayed using μCT. However, the overlaid manual drawings are a viable solution to this problem, combining the benefits of both the historical and modern taxonomic technique.

In an earlier version of this manuscript we argued that the scans provided in this study do not qualify as 3D tomography-based “cybertypes” as hypothetically defined by Faulwetter et al. [15]. Some studies [9,81] selected cybertypes for invertebrate species that were described through the aid of μCT derived images. However, the International Code of Zoological Nomenclature (ICZN) [82] does not depict the concept of cybertypes and after reconsideration and comments by one reviewer, we agree that the notion of high quality 3D images as “cybertypes” is potentially misleading because principally, and no matter how informative, they are not different to (for example) photographs of the actual type specimen. Therefore, instead of referring to them as “types”, for the species description of *P*. *fraserorum* n. sp. the μCT data are referred to as ‘3D type data’, making clear that they are derived information from the physical specimen.

Nevertheless, this present study introduces a holistic species description of a pagurid hermit crab, linking conventional taxonomic practices with DNA molecular barcodes, as well as μCT data that are publicly available for download. Moreover and for the first time, μCT 3D images are combined with line drawings to show high-resolution surface structures in parallel with fine characters and setation. With the application of μCT scanning taxonomic science has progressed into a modern era [9], in which this technology can also advance species descriptions of decapod crustaceans. We showed that hermit crabs, and certainly most other Crustacea, are suitable candidates for this now well-established method to be applied in taxonomy. Whether one of the main benefits of μCT, which is the sharing of 3D data of valuable specimens, will advance and streamline information exchange and to some extent replace the difficult process of loaning physical specimens, however, has to be explored further. This might only happen when the majority of taxonomic experts have access to scanning facilities, as well as to the necessary software for processing 3D μCT data.

## Supporting information

S1 Fig Interactive three-dimensional, μCT derived volume reconstruction of *Pagurus fraserorum* n. sp. male holotype (SAMC MB-A066790). Note that only well-calcified structures are illustrated, as scanning limitations and data processing to remove scanning artefacts reduced the possibilities of visualizing soft body parts (pleon, posterior carapace, membranous structures like gills and setation). Small, calcified characters like tiny spines are also not displayed in this low-resolution version. Clicking on the image activates the interactive 3D-mode, allowing the viewer to move, rotate and zoom into the character of choice.
(STL)

S1 Video *Pagurus fraserorum* rotation movie female paratype. *Pagurus fraserorum* **n. sp.** 3D reconstruction of female paratype, wrapped and scanned in parafilm; scanning artefacts and ‘noise’ not removed, leaving majority of setation, parts of the pleon and the eggs carried on the pleopods.
(MP4)

S2 Video *Pagurus_fraserorum* rotation movie male holotype right cheliped. *Pagurus fraserorum* **n. sp.** 3D reconstruction of right cheliped of male holotype, isosurface rendering.
(MP4)

S3 Video *Pagurus_fraserorum* rotation movie male holotype left cheliped. 3D reconstruction of left cheliped of male holotype, isosurface rendering.
(MP4)
